# Case report: Sandwich therapy of CAR-T combined with ASCT: Sequential CAR-T cell therapy with ASCT after remission with CAR-T therapy caused long-term survival in a patient with relapsed/refractory Burkitt’s lymphoma with TP53 mutations

**DOI:** 10.3389/fimmu.2023.1127868

**Published:** 2023-03-17

**Authors:** Qi Zhang, Xiaojian Zhu, Bo Liu, Yicheng Zhang, Yi Xiao

**Affiliations:** ^1^ Department of Hematology, Tongji Hospital, Tongji Medical College, Huazhong University of Science and Technology, Wuhan, Hubei, China; ^2^ Department of Oncology, Tongji Hospital, Tongji Medical College, Huazhong University of Science and Technology, Wuhan, Hubei, China

**Keywords:** Burkitt’s lymphoma, TP53 mutations, CAR-T, ASCT, relapsed/refractory

## Abstract

Burkitt’s lymphoma (BL) with TP53 mutation often has poor outcome after standard chemoimmunotherapy. Adoptive chimeric antigen receptor (CAR)-T cell therapy may be a new paradigm for treating refractory/relapsed (r/r) BL, but its therapeutic effects remain inconclusive. Here, we report a patient with r/r BL who failed to achieve complete remission (CR) and progressed rapidly after multiple protocol chemotherapy. The patient achieved CR with CAR19 and CAR22 T-cell cocktail therapy and obtained long-term disease-free survival after autologous hematopoietic stem cells (ASCT) and subsequential CAR19 and CAR22 T-cell cocktail therapy. The clinical evolution and genetic features of this case may provide some guidance for CAR-T therapy in overcoming relapses associated with TP53 gene mutations.

## Introduction

Burkitt’s lymphoma (BL) is a highly aggressive B-cell non-Hodgkin’s lymphoma (NHL) that occurs in adults and children. It is classified into local, sporadic, and immunodeficiency-related subtypes, with high extranodal involvement and rapid tumor progression, resulting in a high mortality (1), characterized by MYC oncogene translocation-mediated significant tumor proliferation. With the extensive clinical application of second-generation gene sequencing (NGS), more gene mutations have been detected. Among them, the p53 protein-encoding human TP53 gene located in the short arm of chromosome 17 is an important tumor suppressor and an important factor in DNA repair, cell cycle arrest, apoptosis, aging, and autophagy processes (2). TP53, a common mutant gene in human malignant tumors, is present in blood malignant tumors and various solid tumors, and usually causes abnormal karyotypes. Its missense and nonsense mutations are the driver mutations and prognostic factors in many tumors, and directly affect the cell cycle process (3), which is related to disease progression and poor prognosis. BL with TP53 mutation often has poor outcome after standard chemoimmunotherapy. In principle, the prognosis worsens with the increasing number of tumor gene mutations. However, once patients carry TP53 mutation, the prognosis is extremely poor irrespective of the other mutations they carry. Moreover, the clinical manifestations of patients with TP53 mutation are similar to the characteristics of gene mutation, and there is no significant difference in the prognosis of combined mutation, suggesting that TP53 mutation is the decisive factor.

There is no consensus in the rules of ASCT and CAR-T cell therapy for r/r BL patients till date. Here, we present a patient who failed multiple linear therapy with five gene mutations and expect to provide insights into the therapeutic strategy for such patients.

## Objective

To describe successful sandwich therapy (CAR-T cell therapy is first used to control disease progression and improve patient’s condition, followed by ASCT and subsequential CAR-T cell therapy) of refractory BL with TP53 gene mutations, in order to provide a reference for the treatment of this kind of patients.

## Case report

**Figure 1:**
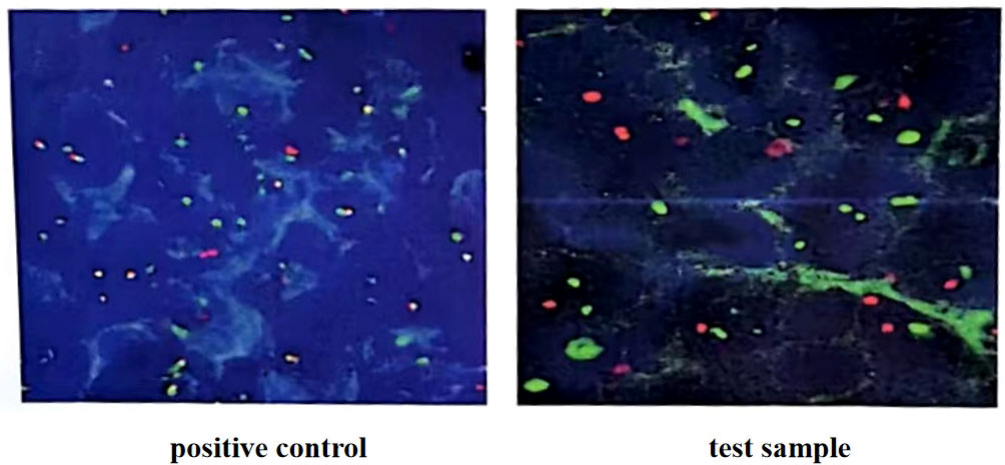
C-myc gene, IgH/BCL2 gene and BCL6 gene translocation detection (in the 100 tumor cells counted, more than 15% of the separation signals were interpreted as positive). Fluorescence *in situ* hybridization confirmed the presence of MYC, while BCL2 and BCL6 genes were negative.

**Figure 2:**
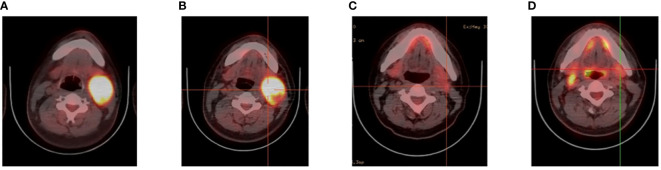
Videography results of relapsed/refractory Burkitt’s lymphoma with TP53 mutations. **(A)** PET before CAR T cell therapy. **(B)** PET after CAR T cell therapy. **(C)** PET after CAR19 and CAR22 T cell cocktail therapy following ASCT. **(D)** The latest PET.

**Figure 3:**
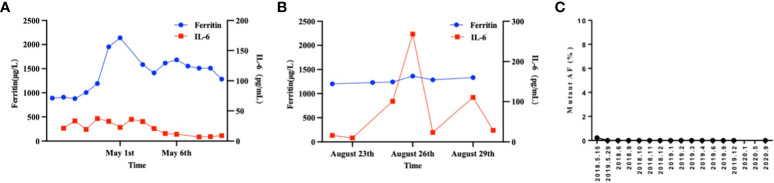
The therapeutic response of CAR19 and CAR22 T cell cocktail therapy following ASCT. **(A)** Dynamic changes in IL-6 and ferritin after CAR-T cell infusion. **(B)** Dynamic changes in IL-6 and ferritin after CAR19 and CAR22 T cell cocktail therapy following ASCT. **(C)** Liquid biopsy results. The proportion of circulating tumor DNA mutations at locus TP53 c.376-1G>A was measured and the results were expressed as the ratio (percentage) of copy numbers of the target gene and the internal reference gene.

**Figure 4:**
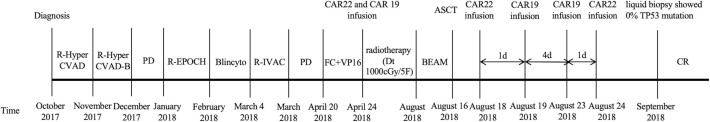
The timeline of the clinical treatment and disease status.

A 26-year-old patient presented to a local hospital in August 2017 with a thumb-sized lump in the left neck for 7 months. The patient was diagnosed with IV stage BL (**Figure 1**). The morphology and immunohistochemistry from a lymph node biopsy were compatible with that of BL. The patient was treated with intensified induction chemotherapy of one cycle of R-HyperCVAD (rituximab, cyclophosphamide, vincristine, doxorubicin, and dexamethasone alternating with high-dose methotrexate and cytarabine), then received one cycle of R-HyperCVAD-B, and transferred to another hospital for treatment in 2018. However, the disease progressed to refractory BL stage II. After R-EPOCH (etoposide, prednisone, vincristine, cyclophosphamide, doxorubicin, and rituximab) treatment and a period of anti-CD19 bispecific T-cell engaging antibody (Blincyto) treatment, the tumor basically disappeared. This was followed by a chemotherapy regimen with R-IVAC (rituximab, ifosfamide, etoposide, and high dose cytarabine). However, two weeks later, the patient found that the tumor grew again. The patient visited our hospital in March 2018 for further diagnosis and treatment, diagnosed as BL stage IIA (relapsed/refractory). Egg-sized swollen masses with slightly hard texture were observed in the left neck. Positron-emission tomography and computed tomography (PET–CT) before treatment demonstrated that the metabolism of the soft tissue mass in the left submandibular region increased, which was considered as lymphoma infiltration (Lugano 5-PS score: 5 points) (**Figure 2A**). Fluorescence *in situ* hybridization confirmed the presence of MYC, while BCL2 and BCL6 genes were negative. Next-generation sequencing identified TP53 mutation, exon 1 of ID3 gene, exon 12 of DDX3X gene, exon 2 of MYC gene, and PHF6 gene. After FC (50 mg fludarabine and 1600 mg cyclophosphamide) combined with 100 mg VP16 pretreatment for three days, CAR-T cells began to be infused on April 24 (6*10^^6^/and 2.3*10^^6^//kg for CD22 and CD19, respectively). After sequential infusion of anti-CD19 and anti-CD22 CAR-T cells, cytokine release syndrome (CRS) was observed, and interleukin-6 and ferritin increased slightly and transiently (**Figure 3A**). Continuous renal replacement therapy (CRRT), plasma change and high-dose methylprednisolone pulse therapy was performed for severe CRS (Grade 4). After infusion for 10 days, the neck mass was substantially reduced and the texture softened. PET-CT showed that the metabolism of soft tissue masses in the left submandibular region reduced, suggesting that some lymphoma infiltration activities were inhibited after treatment (**Figure 2B**). Compared with that observed in the previous PET-CT image (April 3, 2018), the lesion volume and metabolism were significantly reduced. The functional status score (KPS) was 70%, and swollen lymph nodes were palpable in the left neck. Complete remission (CR) was confirmed by PET-CT. After being transferred to oncology department for left neck sensitization radiotherapy (Dt 1000cGy/5F), the KPS was 80%, but no obvious swollen lymph nodes were found in the left neck. On August 9, BEAM regimen pretreatment was performed. Autologous hematopoietic stem cells (2.205*10^^6^/kg CD34^+^ cells) were infused on August 16 and CD19 (1.31*10^6/kg) and CD22 (2.25*10^6/kg) CAR-T cells were infused in two times. The two infusion doses of CD22 CAR T were 1.70*10^6/kg and 0.55*10^6/kg, respectively (August 18 and 24, 2018). The two infusion doses of CD19 CAR T were 0.67*10^6/kg and 0.64*10^6/kg, respectively (August 19 and 23, 2018). CRS was also observed during this period (**Figure 3B**). Re-examination of bone marrow cytology in September showed that megakaryocyte hyperplasia reduced bone marrow picture. No obvious abnormality was found by flow cytometry. Peripheral blood lymphocyte subsets suggest that CD19^+^B cells are 0%; liquid biopsy showed 0% TP53 mutation (**Figure 3C**). In November, the PET-CT showed a little flaky soft tissue shadow in the left submandibular area, and the metabolism did not increase (**Figure 2C**). It was considered that the activity of lymphoma infiltrating focus was substantially inhibited after treatment. Compared with those in the previous PET-CT images (May 30, 2018), the lesion volume and metabolism reduced substantially (Lugano 5-PS score: 1 point). The patient was then reviewed regularly. PET-CT (April, 2019) showed a little patchy soft tissue shadow in the left submandibular area and no increase in metabolism (Lugano 5-PS score: 2 points). Small lymph node shadows were visible in bilateral cervical regions I–II and individual metabolism was slightly higher on the right side. A small retroperitoneal lymph node shadow was visible, with no increase in metabolism. The possibility of inflammatory or non-specific lymph nodes was considered. Then, the latest PET/CT showed the size of the lymph nodes in the right cervical region II was similar, and metabolism reduced slightly, compared with the previous PET/CT images (December 17, 2019) (**Figure 2D**). The timeline of the clinical treatment and disease status were shown in **Figure 4**.

Through telephonic follow-up, we confirmed that the patient is in good condition and has a disease-free survival period of more than four years. When asked about the patient’s feelings from the diagnosis to the complete remission, the patient expressed excitement and despair at the moment of diagnosis and the pain of later treatment, until he came to our hospital to see a glimmer of hope. The patient is incredibly grateful for the help of doctors and the support of his families, especially his father. Now he is in good condition and is actively starting a new life.

## Discussion

TP53 usually acts as a tumor suppressor gene, but is often inactivated by missense mutation in the DNA-binding domain, which causes protein expansion, decreased thermal stability, and DNA binding and transcription function loss (4). TP53 mutations accelerate genomic instability and cancer cell evolution, and support their growth and survival (5). They also mediate interferon (IFN) downregulation, inhibit exogenous apoptotic pathways, and induce immunosuppressive TME. Notably, these mechanisms mediate CAR-T cytotoxicity. TP53 mutations are associated with dysregulation of CAR-T cytotoxicity-related pathways, including IFN and death receptor signaling pathways (6). As an intrinsic tumor factor, TP53 leads to poor prognosis and potential drug resistance in BL. In summary, the high recurrence rate and early recurrence in patients with TP53 mutations may be due to accelerated mutation, increased tumor cell proliferation or viability, and/or interference with CAR-T cell killing (7). In addition, the TP53 mutant circulating tumor DNA (ctDNA) is a potential tool for identifying the response effect of CAR-T cells in NHL patients after treatment. In the early stage, the TP53 mutant ctDNA can predict disease progression and PFS of patients, and its level can also reflect the tumor burden change (8). Therefore, for patients with TP53 mutation, the TP53 mutant ctDNA may be monitored.

In this case, the patient was critical during admission and in poor general condition while the tumor was progressing rapidly. Therefore, the key point of sandwich therapy is to adopt CAR-T cocktail therapy first and ASCT combined with sequential CAR-T cocktail therapy was performed after CR was achieved and the general condition improved. This is because if ASCT combined with sequential CAR-T cocktail therapy was directly performed, the effect might be poor; moreover, the extremely poor physical condition might increase the risk of patient death during transplantation. In addition, the double-targeted CAR-T cocktail therapy can prevent tumor antigens from escaping and reduce CD19 negative relapse, particularly in TP53 mutations. So, the above sandwich therapy of CAR-T combined with ASCT scheme was finally adopted, which was relatively safe and effective.

In conclusion, many studies have recently revealed TP53 mutations as independent prognosis indicators for patients treated with standard chemotherapy. BL patients with a TP53 mutation may not benefit from intensive chemotherapy, ASCT alone, or other targeted therapy strategies. CAR-T therapy alone is also ineffective for BL patients with poor prognosis. A study evaluated the efficacy and safety of CAR 19/22 T-cell therapy for six patients with refractory BL with poor prognostic factors, among which three patients finally achieved objective remission (3/6 50%) (9). However, when ASCT was further used in combination with sequential infusion of CAR19 and CAR22 T cells, the response and survival in TP53-disrupted B cell lymphoma, even in patients with SD/PD prior to transplantation, was good. ASCT and CAR T cell therapy have potential synergistic effects (10). An open-label single-arm prospective clinical study at our center demonstrated that ASCT and sequential CD19/CD22 CAR T-cell cocktail therapy showed high CR rate and good safety in R/R aggressive B-NHL which was ineffective to chemotherapy (11). CAR-T therapy can overcome genetic adverse characteristics to induce remission, including TP53 mutations (12). CAR19 and CAR22 T cell cocktail therapy following ASCT is expected to achieve a long-term survival effect in BL patients with TP53 mutation (13). For high-risk BL patients with TP53 mutation or patients with early relapse and poor prognosis, CAR19 and CAR22 T cell cocktail therapy should be first used to control disease progress, and then CAR19 and CAR22 T cell cocktail therapy following ASCT should be applied, or attempt to selectively consolidate allo-HSCT after CAR-T cell therapy, which may be a direction worth exploring.

## Data availability statement

The original contributions presented in the study are included in the article/supplementary material. Further inquiries can be directed to the corresponding authors.

## Ethics statement

The studies involving human participants were reviewed and approved by Medical Ethics Committee of Tongji Hospital, Tongji Medical College, Huazhong University of Science and Technology (TJ-IRB20160310). The patients/participants provided their written informed consent to participate in this study. Written informed consent was obtained from the patients/participants for the publication of this case report.

## Author contributions

QZ analyzed the data and wrote the manuscript. XZ, BL, and YZ directed the research. YX revised the manuscript. All authors contributed to the article and approved the submitted version.
